# Vocal process granuloma: clinical characterization, treatment and evolution

**DOI:** 10.1016/S1808-8694(15)31205-2

**Published:** 2015-10-20

**Authors:** Elza Maria Lemos, Luiz Ubirajara Sennes, Rui Imamura, Domingos H. Tsuji

**Affiliations:** 1 Otorhinolaryngologist, Assistant physician.; 2 Full Professor, Associate Professor, Discipline of Otorhinolaryngology, Medical School, USP. Director, Service of Bucopharyngology, Clinical Division of Otorhinolaryngology, Hospital das Clínicas, Medical School, USP.; 3 Assistant Physician, Ph.D., Division of Clinical Otorhinolaryngology, Hospital das Clínicas, Medical School, USP.; 4 Full Professor, Associate Professor, Discipline of Otorhinolaryngology, Medical School, USP. Study conducted at the Division of Clinical Otorhinolaryngology, Hospital das Clínicas, Medical School, USP.

**Keywords:** granuloma, vocal fold, vocal process

## Abstract

Vocal process granuloma is a disease whose etiopathogenesis is not well defined. Therefore, its clinical and surgical treatment is not standardized and its therapeutic results depend on the hospital where it is seen. **Aim:** Aiming to characterize patients with vocal process granuloma treated in our hospital, the therapeutic approach used and clinical evolution. **Material and method:** We performed a retrospective review of records. **Results:** We found more male vocal process granuloma, except when associated with laryngeal intubation. The most frequent related etiopathogenic factor was laryngeal-pharynx reflux, followed by laryngeal intubation and vocal abuse. Clinical management with proton-pump inhibitor (PPI), topical inhalant steroid and phonotherapy was enough for remission on 48.6% of the patients. Surgery for removal of the granuloma associated with clinical management was effective in 90% of the events. Later recurrences (more than one year) were noticed in five patients, suggesting that associated etiopathogenic factors should be held for a long time.

## INTRODUCTION

Vocal process granuloma is a nonspecific inflammatory process formed by granulation tissue that occurs primarily in the vocal process of arytenoid cartilage ^1^.

Etiopathogenesis of granuloma is still undetermined and it is attributed to three predisposing factors: vocal abuse, laryngo-pharyngeal reflux disease (RLF) and laryngeal intubation. If none of these causes are found, it is considered idiopathic. It is predominant in male subjects, except in cases associated with laryngeal intubation, which has higher incidence in female cases.

Treatment is initially clinical and surgery is indicated in persistent cases. The type of clinical and surgical treatment has not been standardized yet between different services and, consequently, the therapeutic results are variable.

The purpose of the present study was to characterize patients with vocal process granuloma treated in our service, to present a therapeutic protocol used and to assess the clinical progression of these patients.

## MATERIAL AND METHOD

We conducted a retrospective study in which we reviewed the medical charts of all patients seen in the Group of Voice, HCFMUSP, between May 1996 and May 2003, with diagnosis of vocal process granuloma. We excluded all cases of suspected malignancy and anterior commissure granulomas or other regions of the vocal folds, such as the postoperative cases of laryngeal microsurgery using laser CO_2_.

Data concerning gender, age and main complaint of the patients, key associated etiopathogenic factors, treatment performed and progression were obtained from the medical charts. All patients underwent videotelelaryngoscopy before and after each treatment, with follow up that ranged from 3 to 55 months, mean of 15.2 ± 15.9 months.

The statistical methodology included chi-square test and likelihood ratio to check the association between categorical variables. We considered as significant values p < 0.05.

## RESULTS

We studied 55 patients with diagnosis of vocal fold granuloma. Out of the total, 38 (69%) were male, 17 (31%) were female. Ages in male patients ranged from 21 to 85 years, mean of 48.9 years ± 15.4 years, female subjects ranged from 19 to 64 years, mean age of 40.4 years ± 14.0 years.

**Graph 1:**
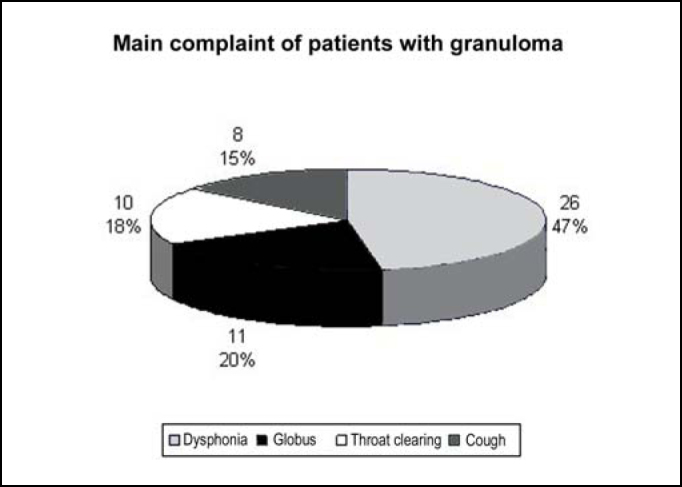
Main complaint in patients with granuloma.

The main complaints of patients are presented in Graph 1. The duration of complaints ranged from 20 days to 24 months, mean duration of 4.5 ± 4.6 months.

As to etiopathogenic factors, 20 people reported symptoms (hoarseness, globus pharyngeal, chronic cough, throat clearing, odynophagia) and/or presented signs (hyperemia and edema of arytenoid region, interarytenoid pakidermia and subglottic edema) suggestive of RLF, 14 patients referred history of laryngeal intubation related with onset of symptoms, 7 patients presented classical history of vocal abuse (for some reason, they had used their voice with louder intensity, inappropriately or had screamed), and 14 were considered of idiopathic cause. Table 1 shows the main etiopathogenic factors identified and their distribution by gender.

**Table 1:** Key etiopathogenic factor and distribution by gender.

	Gender	
Etiopathogenic Factor	Male	Female
RLF (n = 20)	18 (90.0%)	2 (10.0%)
Intubation (n = 14)	6 (42.9%)	8 (57.1%)
Vocal abuse (n = 7)	4 (57.1%)	3 (42.9%)
Idiopathic (n = 14)	10 (71.4%)	4 (28.6%)

All patients were initially submitted to common empirical clinical treatment, regardless of identified etiopathogenic factors. The treatment was based on use of proton pump inhibitors (normally omeprazole 20-40mg/day), inhaled corticoids (normally beclomethasone 750µg/day), and speech therapy (one session a week) for 2 to 4 months. Those that presented improvement of symptoms and disappearance of granuloma were discharged. In those that maintained the granuloma, surgical treatment was indicated. Surgical treatment was conducted with removal of granuloma (cold or CO_2_ laser) associated or not with type A botulinium toxin (Botox) in the thyroarytenoid muscle, as previously described ^2^. In the postoperative period, patients were submitted to clinical treatment including the use of antibiotics, proton pump inhibitor, inhaled corticosteroids, and eventually, speech therapy to reduce the risk of recurrence.

Out of 55 patients who were initially assessed, 20 (36.4%) abandoned treatment after the first visit. Among the patients that did not carry on with clinical treatment, 14 (70%) were male and 6 (30%) were female. There was no association between interruption of treatment and gender (p = 0.912). We did not observe association between interruption of treatment and etiopathogenic factor given that among the patients that abandoned therapy 7 cases (35%) were caused by RLF and 6 (30%) were idiopathic, 5 (25%) were caused by intubation and 2 (10%) by vocal abuse (p = 0.928).

Thus, 35 patients aged between 19 and 76 years, mean of 46.4 + 14.6 years, were reassessed after clinical treatment, and 24 (68.6%) were male and 11 (31.4%) were female. The identified etiopathogenic factor in 13 (37.1%) of the cases was RLF, 9 (25.7%) had had laryngeal intubation, 5 (14.3%) vocal abuse and 8 (22.9%) were considered idiopathic.

Out of 35 reassessed patients, 17 (48.6%) presented remission of granuloma after clinical treatment. The distribution of remissions in relation to identified etiopathogenic factor may be seen in Table 2. As we could observe, in 80% of the cases associated with vocal abuse, there was remission as a result of clinical treatment.

**Table 2:** Distribution of remissions after clinical treatment in different etiopathogenesis.

	Remission after clinical treatment	
Etiopathogenic factor	Yes	No
RLF (n = 13)	6 (46.2%)	7 (53.8%)
Intubation (n = 9)	4 (44.4%)	5 (55.6%)
Vocal abuse (n = 5)	4 (80.0%)	1 (20.0%)
Idiopathic (n = 8)	3 (37.5%)	5 (62.5%)

Likelihood ratio: p = 0.459

Out of 18 patients that persisted with the lesion and were submitted to surgical treatment, 16 (88.9%) presented remission. Early recurrence (after 3 months) after surgery was observed in 2 patients, both submitted to simple excision, without application of Botox. The etiopathogenic factors were RLF and idiopathic cause, respectively. They were submitted to a new surgery with application of Botox, presenting remission of the pathology (Table 3).

**Table 3:** Follow up and progression of patients treated of vocal process granuloma.

Etiopathogenic factor	Remission before surgical treatment	Remission after surgical treatment	Early recurrence
RLF (n = 13)	6 (46.2%)	6 (46.2%)	1 (7.6%)
Intubation (n = 9)	4 (44.4%)	5 (55.6%)	
Vocal abuse (n = 5)	4 (80.0%)	1 (20.0%)	
Idiopathic (n = 8)	3 (37.5%)	4 (50.0%)	1 (12.5%)

Five patients presented late recurrence after a period of remission that ranged from 15 to 51 months, with mean of 37.2 + 16.5 months, three in the same site as the initial lesion, one contralateral to the primary lesion and one that used to be bilateral was recurred as unilateral lesion. New clinical treatment was performed and 80% (4) of the patients reached remission of granuloma.

## DISCUSSION

The etiopathogenesis of vocal fold granuloma remains undetermined ^3^. It is an affection with higher incidence in men, despite the fact that there is no clear explanation about this fact. The frequency, however, is inverted when we consider only the case of post-intubation granuloma, in which women are more prevalent, as demonstrated in this study. Considering the relation between intermembrane and intercartilaginous portions (glottic portion) of the vocal fold in men (adduction = 1.2) than in women (adduction = 1.0), we may suggest that the female larynx is more susceptible to traumas by laryngeal intubation in the posterior region ^4^.

The vocal process granuloma occurs in the cartilaginous portion of the glottis, not interfering with the membranous portion and thus, it does not normally lead to dysphonia, except in cases of large volume lesions. The main complaint of patients in our study was dysphonia. Given that patients were seen in the Group of Voice of our hospital, there may have been a bias in the selection of cases because the referred patients had predominance of vocal symptoms. Moreover, among the etiopathogenic factors of granuloma, we can include RLF and vocal abuse, found in 37% and 13% of our patients, respectively. These factors may lead to dysphonia per se and may have contributed to our findings.

Posterior laryngeal aggressive factors are considered to be predisposing factors in onset of granuloma 5, 6. Acid exposure of the pharynx is prevalent in cases of granuloma. According to Ylitalo (2002), acid reflux on the pharynx (detected by 24-hour pH meter) occurred in 17 out of 26 cases with granuloma (1 to 20 episodes per patient) and in 5 out of 19 controls (1 to 8 episodes per person). The duration of reflux is short and predominant in the standing position.

As to post-intubation granulomas, there is no correlation between time of intubation and occurrence of granuloma. Upon assessing the larynx of post-orotracheal intubation cases, Santos (1994) observed: laryngeal erythema in 94%, ulceration in 76%, with resolution within 6 weeks. Laryngeal granuloma was detected in 44% of the cases and most granulomas (57%) were developed four weeks after extubation ^7^.

Patients with vocal abuse present low pitch, monotone voice, abuse of vocal fry, and hyperfunction, which would lead to further trauma in the vocal processes during phonation ^8^.

Clinical treatment of vocal process granuloma, according to the literature, may include empirical treatment for RLF, inhaled corticoids and speech therapy 6, 9, 10, 11. As to control of RLF, treatment with proton pump inhibitor (PPI) is more efficient than anti-histaminic H2 ^12^. Treatment with inhaled corticoids has good response in granulomas, and it may be the first treatment before surgical intervention ^13^. Speech therapy reduces hyperfunction, elevates the pitch up to comfortable level and increases the number of vocal inflections. These measures reduce the aggression to the arytenoid vocal process, allowing improvement of 46.7% of patients with granuloma 5, 6, 8, 11.

In our study, the advocated clinical treatment initially used focused on empirically treat RLF and vocal abuse, regardless of identified etiopathogenic factors. We decided to follow this approach because many times these factors might be associated and it is difficult to exclude the possibility of a second factor being present in a specific patient. Thus, there was the possibility of having a second factor present in the patients. Therefore, even patients with granuloma associated with laryngeal intubation were treated with PPI, topical corticoids and speech therapy. Despite comprehensive treatment, the index of cure was relatively low (about half of the cases). The insistence in clinical treatment for longer periods of time than four months could have improved our rates. According to Koufman (1994), the resolution of granuloma with clinical treatment may take as long as six to eight months.

When the granuloma is refractory to clinical treatment, surgery is indicated 6, 14, 15. However, surgery should be associated with clinical treatment also in the postoperative period to reduce the risk of recurrence.

As to surgical treatment, we did not observe evident differences between the groups, but we point out that 5 out of 5 patients (100%) operated of granuloma associated with intubation presented remission of the lesion.

In past years, the use of Botox has been supporting surgical treatment to reduce recurrences. The injection is made in the thyroarytenoid muscle and works as (1) inhibitor of hypertonicity; (2) strengthens antagonist muscles, and (3) restore balance of forces 6, 14, 15.

As a result of the reduction of the adduction force of the vocal fold by the action period of Botox, there is no forced contact between vocal processes during phonation, cough and throat clearing, allowing scaring of mucosa and disappearance of granuloma.

Early recurrence (before 3 months of surgery) in our study was below that reported in the literature 3, 9, 16. Only 2 patients that underwent surgical treatment without Botox had recurrence of granuloma.

The cases of late recurrence (after one year) have been rarely described in the literature. The follow up of these patients for a more prolonged period of time showed that some patients still present the same initial symptoms and there was re-onset of granuloma. None of the cases was associated with post-intubation granuloma. Out of 5 cases that presented late recurrence, three reported RLF symptoms. In this period, they had not used anti-reflux drugs. It is possible that by maintaining the reflux disease out of control, there would be a new granuloma. These data are still scarce and have to be properly studied, but they suggest that patients with RLF that develop vocal fold granuloma should be maintained under clinical treatment for more prolonged periods of time.

A fact that attracted our attention in the study was that about one third of the patients abandoned the follow up after the first visit. In this visit, they were instructed about the nature of the laryngeal lesion and submitted to the described clinical treatment. It is possible that many of them have improved their symptoms with treatment and then abandoned the follow up. Giving up treatment after the first visit makes us wonder whether we should instruct further the patient about the importance of treatment, disappearance of granuloma and differential diagnosis. The vocal fold process is conceptually a benign lesion. However, differential diagnosis includes malignant laryngeal lesions. Thus, in selected cases (such as atypical location of lesions and progressive growth despite treatment), we should make a biopsy to be absolutely sure about the nature of the lesion ^10^. Luzar (2000), in a clinical and histomorphological retrospective study of 149 biopsies of laryngeal granulomas found simple hyperplasia in 65.8% (98 patients), abnormal hyperplasia in 4.7% (7 patients), atrophic epithelium in 16.1% (24 patients), and normal squamous epithelium in 13.4% (20 patients).

## CONCLUSION

In our sample, vocal process granuloma affected mainly men, except for cases associated with laryngeal intubation. The main etiopathogenic factor associated was RLF, followed by laryngeal intubation and vocal abuse. Clinical treatment was based on the use of proton pump inhibitor, topical corticoid and speech therapy that presented efficacy over the lesion in about half of the cases. Surgical treatment associated with clinical treatment was effective in remission of granuloma in about 90% of the cases. Late recurrences (after one year of treatment) may occur, suggesting that follow up and, possibly, control of etiopathogenic factors in the long run are recommended for these patients.
